# Prognostic Impact of Sarcopenic Obesity after Neoadjuvant Chemotherapy Followed by Surgery in Elderly Patients with Esophageal Squamous Cell Carcinoma

**DOI:** 10.3390/jcm9092974

**Published:** 2020-09-15

**Authors:** Sachiyo Onishi, Masahiro Tajika, Tsutomu Tanaka, Keisaku Yamada, Tetsuya Abe, Eiji Higaki, Takahiro Hosoi, Yoshitaka Inaba, Kei Muro, Masahito Shimizu, Yasumasa Niwa

**Affiliations:** 1 Department of Endoscopy, Aichi Cancer Center Hospital, 1-1 Kanokoden, Chikusa-ku, Nagoya 464-8681, Japan; soonishi@aichi-cc.jp (S.O.); tstanaka@aichi-cc.jp (T.T.); k.yamada@aichi-cc.jp (K.Y.); yniwa@aichi-cc.jp (Y.N.); 2 Department of Gastroenterological Surgery, Aichi Cancer Center Hospital, 1-1 Kanokoden, Chikusa-ku, Nagoya 464-8681, Japan; tabe@aichi-cc.jp (T.A.); ehigaki@aichi-cc.jp (E.H.); thosoi@aichi-cc.jp (T.H.); 3 Department of Diagnostic and Interventional Radiology, Aichi Cancer Center Hospital, 1-1 Kanokoden, Chikusa-ku, Nagoya 464-8681, Japan; 105824@aichi-cc.jp; 4 Department of Clinical Oncology, Aichi Cancer Center Hospital, 1-1 Kanokoden, Chikusa-ku, Nagoya 464-8681, Japan; kmuro@aichi-cc.jp; 5 Department of Gastroenterology/Internal Medicine, Gifu University Graduate School of Medicine, 1-1 Yanagido, Gifu 501-1194, Japan; shimim-gif@umin.ac.jp

**Keywords:** elderly patient, esophageal squamous cell carcinoma, neoadjuvant chemotherapy, sarcopenia obesity

## Abstract

We evaluated the impact of body composition on clinical outcomes after neoadjuvant chemotherapy (NAC) followed by surgery for elderly cStage II/III esophageal squamous cell carcinoma (ESCC). Ninety-one patients ≥70 years old and 116 patients <70 years old with ECSS who underwent NAC between January 2013 and June 2018 at the Aichi Cancer Center were included. Body composition as assessed from computed tomography (CT), American Society of Anesthesiologists physical status (ASA-PS), and subjective global assessment (SGA) was assessed before initiation of NAC. Although elderly patients showed significantly poorer ASA-PS (*p* < 0.01) and SGA (*p* < 0.01), and significantly more frequent history of malignancy (*p* < 0.05), no significant differences were identified in the frequencies of adverse events, postoperative complications, or in cancer-specific survival (*p* = 0.65, hazard ratio 1.15), or overall survival (*p* = 0.42, hazard ratio 1.26). However, multivariate analysis identified sarcopenic obesity as the only independent predictor of prognosis in elderly patients. Sarcopenic obesity was associated with higher body mass index (*p* = 0.04), better SGA (*p* < 0.01), and lower pre-treatment weight loss (*p* = 0.03). NAC was as effective and safe for elderly patients without sarcopenic obesity as for young patients. However, diagnosing sarcopenic obesity based on clinical findings is difficult, so the preoperative CT assessment of sarcopenic obesity is important.

## 1. Introduction

Esophageal cancer is the sixth-most common cause of cancer death among men, and the ninth-most common among women worldwide [1]. In Japan, esophageal cancer is more common among men, with 17.9 new diagnoses per 100,000 population annually. The morbidity rate among patients >65 years old is about 66% for esophageal squamous cell carcinoma (ESCC) [2]. Saying that esophageal cancer is a disease of the elderly is thus no exaggeration.

Neoadjuvant chemotherapy (NAC) followed by surgery has been recommended for locally advanced squamous cell carcinoma of the thoracic esophagus following the results of the Japan Clinical Oncology Group (JCOG) trial in Japan [3]. NAC with cisplatin and 5-fluorouracil (CF) followed by surgery is thus considered the most appropriate treatment for locally advanced ESCC. However, the median age of patients enrolled in that trial was 61 years old and all patients were under 75 years old, and thus under the age of the majority of patients treated in the real world. Few reports have examined the safety and efficacy of NAC in the elderly.

In general, Elderly individuals often show an age-related loss of muscle mass, called sarcopenia. In Japan, the prevalence of sarcopenia among the general population over 65 years old has been reported as 20% [4]. Patients with gastrointestinal disease showed a 32% frequency of sarcopenia [5]. The prevalence of sarcopenia was thus predicted to be high in elderly patients with gastrointestinal disease. Sarcopenia, particularly in cancer, has been linked to physical disability, surgical complications, increased risk of severe toxicity during cancer treatment, extended hospitalization, and reduced survival time. In esophageal cancer patients with ESCC, sarcopenia is also reportedly linked with surgical outcomes, chemotherapy or chemoradiation adverse events, and is associated with poor prognosis [6,7,8].

Sarcopenia can occur in any body mass index (BMI) category, from underweight to obese patients, with the latter appearing in a condition known as sarcopenic obesity. Not only sarcopenia, but also sarcopenic obesity has recently been associated with increased risks of postoperative complications, chemotherapy-related toxicity, and decreased survival rates in colon cancer, breast cancer, lung cancer, and pancreatic cancer, among others [9,10,11]. As for esophageal cancer, Anandavadivelan et al. reported that 14% of patients with esophageal cancer had sarcopenic obesity, and reported that not only sarcopenia patients, but also sarcopenic obese patients were at a high risk for developing dose-limiting toxicity during chemotherapy [12]. However, 67 patients in that study had adenocarcinoma. In Japan, the rate of ESCC represents >90% of esophageal cancers, so the rate of esophageal adenocarcinoma is low.

After the onset of esophageal cancer, even obese patients undergo progressive weight loss [13]. Those obese patients who lose muscle mass may also have sarcopenic obesity [14], representing a worst-case scenario due to the combined health risks of obesity and sarcopenia. Sarcopenic obesity has also been reported as an independent predictor of poor prognosis in patients with solid tumor [14]. However, the relationship between sarcopenic obesity and prognosis in patients with ESCC has not been reported. This study examined the safety and efficacy of NAC for the treatment of ESCC in the elderly, who are more likely to have body compositional abnormalities such as sarcopenia and sarcopenic obesity. We also studied the effects of body composition abnormalities on cancer-specific and overall survival (OS) rates.

## 2. Materials and Methods

### 2.1. Study Population

A retrospective cohort study was conducted in 249 consecutive cStage II/III ESCC patients diagnosed and treated at Aichi Cancer Center Hospital, Nagoya, Japan, between January 2013 and June 2018. Patients were excluded if they declined NAC followed by surgery (*n* = 38; 18 elderly, 20 young) or if they had to switch to chemoradiotherapy because of progressive disease (*n* = 4; 2 elderly, 2 young). In total, 207 cStage II/III ESCC patients were included. We investigated 91 ESCC patients ≥70 years old, with 116 patients <70 years old as controls. The World Health Organization defines the elderly as individuals ≥65 years old, whereas our study defined individuals ≥70 years old as elderly. This is because 20% of the elderly in Japan are ≥70 years old, and individuals this age are eligible for the national health insurance. The staging was based on the Guidelines for Diagnosis and Treatment of Carcinoma of the Esophagus 2017 by the Japan Esophageal Society [2]. All patients were examined on the first hospitalization for initial treatment. They conductedcomputed tomography (CT) imaging before treatment fordiagnosis. We approved by the ethics committee of the Aichi Cancer Center Hospital (approval no. 2019-1-563, observational study) and was performed in accordance with the 1975 Declaration of Helsinki, as revised in 1983.

### 2.2. Neoadjuvant Chemotherapy

The final diagnosis and treatment was determined by a conference of gastroenterologists, endoscopists, clinical oncologists, and radiologists at our hospital. Chemotherapy was FP regimen (5-fluorouracil and cisplatin) or DCF regimen (docetaxel, 5-fluorouracil and cisplatin). The FP regimen was administered twice every 4 weeks. Cisplatin was administered at a dose of 80 mg/m^2^ by slow-drip infusion on day 1. Administration of 5-fluorouracil was performed at a dose of 800 mg/m^2^/day by continuous infusion for 24 h on days 1–5. The DCF regimen was repeated three times every 3 weeks, with docetaxel at 70 mg/m^2^ given as a 1-h intravenous infusion on day 1 of each cycle, cisplatin at 70 mg/m^2^ as a 2-h intravenous infusion on day 1 of each cycle, and 5-fluorouracil at 750 mg/m^2^ as a continuous infusion on days 1–5. Under conditions of tumor progression or chemotherapeutic adverse events, each regimen was able to be performed below the scheduled dose (incomplete cases). All patients underwent surgery at least 3–4 weeks after completion of NAC.

### 2.3. Evaluation of Chemotherapy-related Toxicities and Postoperative Complications

Adverse events were assessed according to the Common Terminology Criteria for Adverse Events (CTCAE) version 4.0. Postoperative complications were defined as any complication classified as grade 3 or higher according to the Clavien–Dindo classification.

### 2.4. Measurement and Definitions of Body Composition

Skeletal muscle mass and visceral fat content were measured using CT images taken before treatment. The muscle area at the level of the third lumbar vertebra (L3) is said to correlate with whole body muscle mass [15]. The volume analyzer Synapse VINCENT 3 image analysis system (Fujifilm Medical, Tokyo, Japan) was used to measure the cross-sectional area of skeletal muscle at L3 from abdominal CT. Skeletal muscles were calculated using CT attenuation values with thresholds ranging from −29 Hounsfield units (HU) to 150 HU. To standardize according to patient height, the skeletal muscle mass index (SMI) (cm^2^/m^2^)was calculated by dividint the total skeletal muscle area (cm^2^) at the L3 level by the square of height (m^2^). [16,17]. The anonymized CT images had been analyzed by two trained authors (S.O., Y.I.). Visceral fat was quantified in the range of −200 to −50 HU. Sarcopenia was defined as an SMI index of <42.0 cm^2^/m^2^ in men and <38.0 cm^2^/m^2^ in women, based on the study by Nishikawa et al. [18]. These cutoff values are used in the Japan Society of Hepatology guidelines for sarcopenia in patients with liver disease. Obesity was defined from a visceral fat mass of >100 cm^2^ in both sexes. This cutoff value is widely used for Asian populations to define obesity. We defined sarcopenic obesity as a combination of sarcopenia and obesity.

### 2.5. Patient Data

Clinical data for all patients were collected from the prospectively maintained database at our institution. Height, weight, and BMI were recorded at the time of first hospitalization. Laboratory data were as at first visit to our institution. The general condition at the time of initial admission was assessed using the Americal Society of Anesthetists (ASA)-physical status (ASA-PS). Mortality data were collected via a hospital coding system and by contacting the general practitioner providing treatment for the patient. Mortality data were determined from the date of the first hospitalization until death or the censor date of the study.

### 2.6. Nutritional Screening

In our study, we used the subjective global assessment (SGA) proposed by Baker et al. [19] for nutritional screening; SGA has been reported as a method for predicting the length of hospital stay for patients with gastrointestinal cancer [20]. This examination included a medical history and physical examination component. The medical history consisted of four categories: weight loss, gastrointestinal symptoms, functional capacity, and comorbidities. The physical examination included subcutaneous fat loss, muscle wasting, and edema. Based on the SGA’s comprehensive questions, patients were categorized into three groups: A, adequate nutrition; B, mild to moderate malnutrition; or C, severe malnutrition. SGA was assessed by a registered dietitian for all patients in our hospital during their admission.

### 2.7. Statistical Analysis

The Statistical analysis was conducted using JMP version 9.0.2 software (SAS Institute, Cary, NC, USA). Continuous variables were expressed as mean and ranges, and differences were analyzed using the Mann-Whitney U test. Categorical variables were given as the number of patients and differences in distribution between groups were tested with the chi-square test. Survival curves were evaluated using the Kaplan-Maier method and compared using the log-rank test. Univariate and multivariate Cox regression analysis were performed to examine factors predicting OS in elderly ESCC, and variables that showing *p* < 0.05 in univariate analysis were included in multivariate analysis; statistical significance was declared for values of *p* < 0.05.

## 3. Results

### 3.1. Patient Characteristics

Characteristics of patients from the elderly and younger groups are shown in Table 1. Mean age was 74.1 years (range, 70–81 years) for the elderly group and 64.9 years (range, 60–69 years) for the young. The elderly group had a significantly worse ASA-PS score (*p* < 0.01). In addition, the elderly group was significantly more likely to have diabetes mellitus (*p* = 0.03) and a medical history of malignancy (*p* = 0.01), which included gastric cancer (*n* = 5), prostate cancer (*n* = 5), colon cancer (*n* = 3), pharynx cancer (*n* = 3), cerebral cancer (*n* = 2), and pancreatic cancer (*n* = 2). On the other hand, malignancies in the young included gastric cancer (*n* = 3), colon cancer (*n* = 3), and prostate cancer (*n* = 2). No differences in sex, Brinkman index, or alcohol intake were seen between elderly and young groups. Likewise, no significant differences were identified in tumor factors, such as tumor location or cStage. In terms of nutritional factors, no significant differences in BMI, visceral fat mass, serum albumin, rate of body weight loss during the 6 months preceding treatment, sarcopenia, or sarcopenic obesity were observed between groups. However, total cholesterol was significantly higher in the young group (*p* = 0.02), and the SGA indicated malnutrition (SGA B or C) was significantly more frequent in the elderly group (*p* < 0.01).

Data are expressed as the number of cases or mean ± standard deviation. Malignant disease included gastric cancer (*n* = 8), prostate cancer (*n* = 7), colon cancer (*n* = 6), cerebral cancer (*n* = 2), lung cancer (*n* = 2), hematologic tumor (*n* = 3), lung cancer (*n* = 2), pancreatic cancer (*n* = 2), thyroid gland cancer (*n* = 2), bladder cancer (*n* = 1), breast cancer (*n* = 1), and tongue cancer (*n* = 1).Chronic disease included chronic hepatitis (*n* = 16), chronic obstructive pulmonary disease (COPD) (*n* = 5), chronic kidney disease (*n* = 6), chronic pancreatitis (*n* = 2), chronic thyroiditis (*n* = 1), and rheumatoid arthritis (*n* = 2). ASA-PS, American Society of Anesthesiologists physical status; %VC, percentage vital capacity; %FEV1.0, percentage forced expiratory volume in 1 s; CCr, creatinine clearance; ChE, cholinesterase; T-chol, total cholesterol; Hb, hemoglobin; SGA, subjective global assessment; Ut, upper thoracic esophagus; Mt, middle thoracic esophagus; Lt, lower thoracic esophagus.Body weight loss rate = rate of body weight loss during the 6 months preceding treatment.

### 3.2. NAC-Related Factors

NAC-related factors for the elderly and young groups are shown in Table 2. In the elderly group, 67 patients received the FP regimen and 24 patients received the DCF regimen. In the young group, 72 patients received the FP regimen and 44 patients received the DCF regimen. No significant differences were seen between groups in proportions of chemotherapy regimens or incidence of adverse events ≥ Grade 3.

### 3.3. Operation-Related Factors

Operation-related factors for the elderly and young groups are shown in Table 2. Mean operative time for the elderly and young groups was 497.4 and 478.3 min, respectively. Mean hospital stay was 29.1 and 27.7 days, respectively. No significant differences in either operative time or hospital stay were evident between the two groups. No significant differences in postoperative complications or complications of Clavien-Dindo classification grade ≥3 were evident between groups.

### 3.4. OS Rate

**Figure 1:**
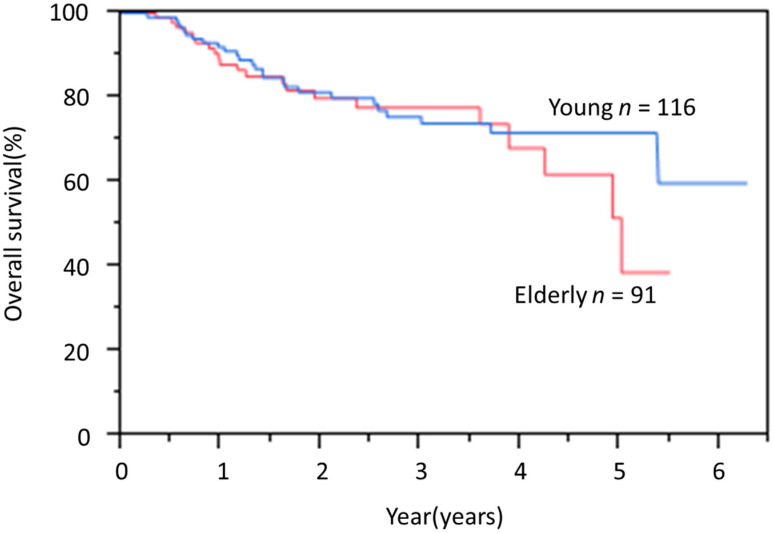
Kaplan-Meier overall survival (OS) curves for elderly and young groups.

The mean duration of follow-up after treatment was 2.5 years. Sixteen patients in the elderly group and four patients in the young group died during follow-up. In the elderly group, 12 patients died of esophageal cancer and four patients died of other diseases. In the young group, two patients died of esophageal cancer and two patients died of other diseases. Likewise, the OS rate showed no significant difference between groups (Figure 1).

### 3.5. Prognostic Factors for OS in the Elderly Group

Univariate analysis of OS showed that sarcopenia and sarcopenic obesity were significantly associated with poor overall survival. Cox proportional hazard regression modeling for OS identified the prevalence of sarcopenic obesity as an independent prognostic factor for poor OS in these patients (hazard ratio (HR), 2.72; 95%, confidence interval (CI) 1.06–7.76) (Table 3).

### 3.6. Characteristics of Sarcopenic Obesity in the Elderly Group

**Figure 2:**
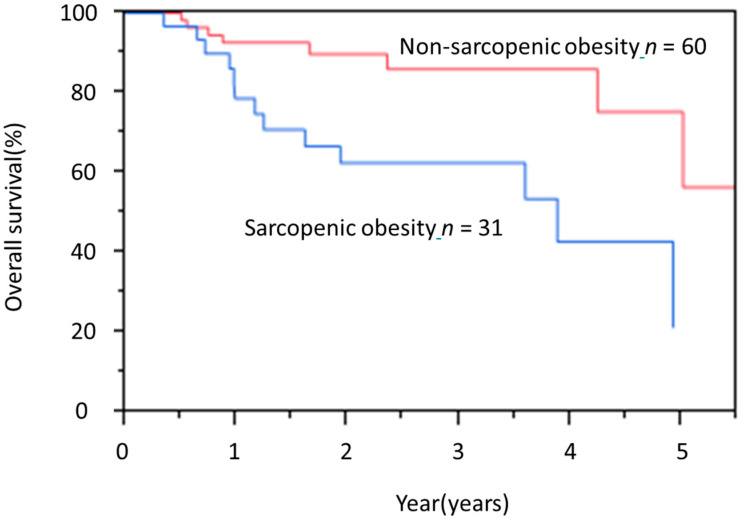
Kaplan-Meier overall survival (OS) curves of elderly patients with and without sarcopenic obesity. HR, hazard ratio; CI, confidence interval.HR, 37.1; 95%CI 1.51–9.88; *p* < 0.01.

Sarcopenic obesity was a complication in 31 patients (34.1%). Sarcopenic obesity in the elderly group was significantly associated with higher BMI, lower rate of body weight loss during the 6 months preceding treatment and SGA indicating good nutrition (SGA A). However, no significant differences in age, sex, ASA-PS, presence of comorbidities, or serum albumin were evident between sarcopenic obesity and non-sarcopenic obesity (Table 4). Similarly, no significant differences in treatment-related factors were noted between these two subgroups. However, pneumonia with Clavien-Dindo classification grade ≥ 3 was significantly more common in sarcopenic obesity (Table 5). The OS rate of patients with sarcopenic obesity was significantly worse than that of patients with non-sarcopenic obesity (Figure 2).

## 4. Discussion

In recent years, the number of elderly patients with cancer has been increasing as life expectancies have increased. In Japan, the incidence of esophageal cancer has gradually increased, and more than 56% of patients with esophageal cancer are >70 years old. Guidelines for the diagnosis and treatment of esophageal carcinoma recommend NAC followed by surgery for patients with cStage II/III esophageal cancer if such a regimen can be tolerated [2]. However, specific descriptions such as age restrictions or correspondence in cases with various comorbidities were lacking from the guidelines. Our study showed that elderly patients could receive NAC followed by surgery with rates of adverse events, complications of surgery, and OS similar to those in younger patients. In addition, our study characterized body composition abnormalities using CT before NAC in patients with ESCC, and we revealed that sarcopenic obesity, but not sarcopenia, is an independent predictor of poor prognosis in elderly patients.

Sarcopenia has been associated with several negative clinical outcomes in cancer. In esophageal cancer, a recent comprehensive systematic review and meta-analysis of patients with postoperative esophageal cancer found that patients with sarcopenia had significantly lower 3- and 5-year OS rates than those without sarcopenia [6,21]. Furthermore, preoperative sarcopenia was identified as an independent predictor of poor OS and disease-free survival (DFS) in surgically treated esophageal cancer patients, regardless of whether the patient received preoperative treatment. However, the significance of sarcopenic obesity, a condition of combined burdens from sarcopenia and obesity, has been less extensively investigated. Obesity is generally evaluated using BMI, but this index cannot account for differences in fat distribution, because direct measurement of adipose tissue is not performed. More recently, CT performed to evaluate the tumor stage in cancer patients has been extensively used for the secondary purpose of body composition analysis. In this study, rates of sarcopenia (75.8%) and sarcopenic obesity (34.1%) tended to be higher in the elderly, but did not show significant differences compared to young patients (62.9% and 23.3%, respectively). The prevalence of sarcopenic obesity in esophageal cancer has remained unclear, with few reports focusing on this issue. Reisinger et al. [22] reported that among patients with esophageal cancer undergoing surgery, the frequency of sarcopenic obesity was 2% using BMI to define obesity or 17% using visceral adiposity to define obesity. Anandavadivelan et al. [12] reported that among patients with cancer of the esophagus or gastric cardia treated using NAC before surgery, the rate of sarcopenic obesity was 14% using visceral adiposity to define obesity. These results were reported from Western countries, where esophageal adenocarcinoma is dominant. In Asian countries, where ESCC is dominant, Sugawara et al. [23] reported that among patients with esophageal cancer undergoing surgery, sarcopenic obesity was rare using BMI to define obesity. Such differences are clearly at least partially attributable to definitions of obesity using either visceral fat mass or BMI, along with ethnic, cultural, and histological differences. The differences between the results from Sugawara et al. and our own findings may be explained by differences in definitions of obesity, and the definition of sarcopenic obesity requiring standardization.

To the best of our knowledge, this is the first report to find sarcopenic obesity, but not sarcopenia, as a factor independently associated with poor prognosis among elderly ESCC patients. A recent review [24] covered the definitions, prevalence, and clinical implications of sarcopenic obesity in medical and surgical oncology. That review showed that sarcopenic obesity is independently associated with higher mortality and a higher rate of complications in systemic and surgical cancer treatment, across multiple cancer sites and treatment plans. In our study, no significant differences in adverse events during NAC were evident, but a higher prevalence of pneumonia after surgery had appeared in sarcopenic obese patients compared with non-sarcopenic obese patients. In esophageal cancer, although Reisinger et al. [22] found no association between sarcopenic obesityand postoperative mortality, Anandavadivelan et al. [12] showed that sarcopenic obese patients may be at higher risk of developing dose-limiting toxicities during chemotherapy compared to non-sarcopenic patients. The underlying mechanisms remain unexplained, but the association between obesity and surgery-related complications has been reported. Obese patients are more likely to suffer from hypertension, diabetes mellitus, and metabolic syndrome [25]. Obesity is also known to have an effect on inflammation and insulin resistance. Some studies [26,27,28,29] have reported that these conditions may impair the immune response to surgical stressand increase the risk of surgical site infection, poor wound healing, and delayed recovery. Because both sarcopenia and obesity are strongly associated with inflammation [30], the metabolic responses may affect surgical outcomes in sarcopenia obesity patients. The association of sarcopenic obesity with toxicity from chemotherapy was hypothesized to involve the fact that chemotherapy doses were adjusted according to body surface area (BSA) in the present study, so the dose of chemotherapy drugs in patients with sarcopenic obesity may have been overestimated. Furthermore, in such patients, responses to the stressors of surgery and chemotherapy may have been further impaired, resulting in increased complication rates and poor prognosis.

Notably, in this study, elderly patients with sarcopenic obesity had a significantly higher BMI, lower levels of malnutrition according to SGA, and lower rates of pre-treatment weight loss. In cancer patient, muscle mass is not correlated with BMI or BSA. It is understood that cancer patients do not necessarily lose or gain fat and skeletal muscle in equal proportions when their weight changes and indeed fat can be gained while muscle is being lost [15,31]. This phenomenon may be prominent in the elderly patients. Recognizing sarcopenic obesity based on regular clinical information is thus difficult. For surgical candidates who routinely undergo preoperative CT, the detection of sarcopenic obesity before treatment is possible and important. In patients with resectable ESCC, NAC before surgery is standard. We apply a period of at least 2 months before surgery, so interventions to improve sarcopenic obesity are required. Weight loss in the perioperative period may be common as a side effect of chemotherapy, postoperative complications and postoperative dietary restrictions. Energy restriction with or without exercise results in a loss of approximately one-quarter of lean mass per unit weight, which could worsen sarcopenia [32]. Paradoxically, supporting obese patients so that they do not lose weight is therefore of particular importance. An integrated approach will include timely dietary counselling and physical activity [12]. For the physical activity, aerobic and resistance exercises are core components in the treatment of sarcopenic obesity, but specific times and intensities should be considered based on the individual case [32]. We provide perioperative rehabilitation by a physical therapist, but do not perform active rehabilitation during NAC because of the short hospital stay. We need to apply therapeutic interventions for the duration of NAC depending on the individual case in the future.

The present study has various limitations that need to be considered when interpreting the study findings. First, this was a retrospective cohort study at a single institution with possible selection bias, because the study was designed for patients with preserved cardiopulmonary function who could undergo surgery. Second, we could not evaluate muscle functions such as grip strength or gait speed. The European Working Group on Sarcopenia in Older People [33] suggested an algorithm for sarcopenia based on measurements of both muscle functional status and mass. However, criteria for sarcopenia in clinical oncology to date have been largely based on CT, which offers the highest available precision and specificity for determining muscle mass, fat mass, and distributions. CT data are readily available in clinical records, because CT is conducted for diagnosis and evaluation of treatment response. Our results will thus be available for elderly ESCC. Third, cutoff values of SMI for sarcopenia were given in the Japan Society of Hepatology guidelines [18] for sarcopenia in patients with liver disease, because a standard SMI cutoff for defining sarcopenia has not been established. The most frequently used cutoff points were published in 2008 by Prade et al. [14], in a population of obese Canadians with lung and gastrointestinal cancers. Asian populations were found to be more prone to central obesity and low skeletal muscle with increased insulin resistance compared to Western populations [34]. The results of this study therefore may not be applicable to other biogeographic and ethnic groups.

## 5. Conclusions

NAC followed by surgery in a real-world setting is an effective, safe treatment in elderly ESCC patients, as in young patients. However, even in elderly patients, sarcopenic obesity was associated with poor prognosis. Because diagnosing sarcopenic obesity based on clinical findings is difficult, the ability to recognize sarcopenic obesity using CT is important.

## Figures and Tables

**Table 1:** Baseline characteristics of the elderly and young groups.

	Total	Elderly	Young	
Variables	(*n* = 207)	(*n* = 91)	(*n* = 116)	*p*
Age (years)	68.9 ± 5.4 (60–81)	74.1 ± 2.9 (70–81)	64.9 ± 2.9 (60–69)	<0.01
Sex (male/female)	169/38	73/18	96/20	0.71
ASA-PS (1/2)	70/137	21/70	49/67	<0.01
Body mass index (kg/m^2^)	21.0 ± 2.8 (13.7–30.5)	21.1 ± 2.8 (13.7–30.5)	20.9 ± 2.8 (14.8–27.3)	0.81
Body mass index >25 kg/m^2^	15 (7.3)	6 (6.6)	9 (7.7)	0.79
Visceral fat mass (cm^2^)	93.9 ± 55.9	94.9 ± 55.9	93.1 ± 56.1	0.81
Obese	91 (44.0)	41 (45.1)	50 (43.1)	0.78
Albumin (g/dL)	4.13 ± 0.37	4.09 ± 0.41	4.16 ± 0.34	0.14
CCr	75.9 ± 15.1	74.2 ± 15.1	77.3 ± 14.9	0.13
ChE	292 ± 64	290 ± 61	294 ± 66	0.63
T-chol	196 ± 36	189 ± 32	201 ± 39	0.02
Hb	13.5 ± 1.5	13.5 ± 1.3	13.6 ± 1.7	0.54
SGA (A/B or C)	149/58	53/38	96/20	<0.01
Body weight loss rate (%)	3.4 ± 5.5	3.4 ± 5.5	3.3 ± 5.5	0.83
Sarcopenia, *n* (%)	142 (68.6)	69 (75.8)	73 (62.9)	0.06
Sarcopenic obesity, *n* (%)	58 (28.0)	31 (34.1)	27 (23.3)	0.08
%VC	101.4 ± 13.3	100.6 ± 15.1	102.1 ± 11.7	0.42
%FEV1.0	77.2 ± 8.0	76.9 ± 7.8	77.4 ± 8.2	0.62
Diabetes mellitus, *n* (%)	32 (15.5)	20 (21.9)	12 (10.3)	0.03
Cardiovascular disorder, *n* (%)	97 (46.9)	49 (53.8)	48 (41.4)	0.09
Cerebrovascular disorder, *n* (%)	2 (0.9)	2 (2.2)	0(0)	0.19
Malignant disease, *n* (%)	40 (19.3)	25 (27.5)	15 (12.9)	0.01
Chronic disease, *n* (%)	32 (15.4)	10 (10.9)	22 (18.9)	0.12
Primary tumor location (Ut/Mt/Lt)	31/109/67	13/48/30	18/61/37	0.93
Clinical T (1/2/3)	35/41/131	16/17/58	19/24/73	0.41
Clinical N (0/1/2/3)	25/109/68/5	12/53/26/0	13/56/42/5	0.11
Clinical stage (II/III)	68/139	29/62	39/77	0.88

**Table 2:** Treatment-related factors in the elderly and young groups.

	Elderly	Young	*p*
Variables	(*n* = 91)	(*n* = 116)	
Adverse events (≥ grade 3), *n* (%)			
Hematological			
Neutropenia	40 (46.0)	69 (59.5)	0.06
Non-hematological adverse events			
Diarrhea	3 (3.5)	7 (6.0)	0.52
Malaise	8 (9.2)	8 (6.9)	0.6
Anorexia	8 (9.2)	8 (6.9)	0.6
Incomplete case	6 (6.6)	8 (6.9)	0.31
Operation time (min)	497.4 ± 96.3	478.3 ± 89.1	0.14
Hospital stay (days)	29.1 ± 17.9	27.7 ± 17.5	0.57
Surgery-related complications (≥Grade 3), *n* (%)	29 (31.9)	42 (36.2)	0.55
Anastomotic	4 (4.4)	7 (6.0)	0.75
Pneumonia	7 (7.7)	3 (2.6)	0.11
Recurrent nerve paralysis	2 (2.2)	1 (0.9)	0.58
SSI	10 (11.0)	12 (10.3)	1.00
Chylothorax	2 (2.2)	2 (1.7)	1.00
Arrhythmia	0 (0)	1 (0.9)	1.00
Anastomosis stenosis	15 (16.5)	27 (23.3)	0.30

Data are expressed as the number of cases or mean ± standard deviation. SSI, surgical site infection.

**Table 3:** Cox proportional hazard model of clinical characteristics of overall survival in the elderly group using uni- and multivariate analysis.

			Univariate		Multivariate	
Variables		*n*	HR (95%CI)	*p*	HR (95%CI)	*p*
Age, years	<75	57	2.11 (0.82–6.16)			
	≥75	34	1	0.12		
Sex	male	73	2.78 (0.80–17.5)			
	female	18	1	0.11		
cStage	III	62	1.47 (0.57–4.49)			
	II	29	1	0.43		
NAC	DCF	24	1.79 (0.71–4.36)			
	FP	67	1	0.21		
ASA-PS	2	70	2.54 (0.73–16.1)			
	1	21	1	0.16		
DM	+	20	2.11 (0.77–5.38)			
	-	71	1	0.14		
Cardiovascular event	+	49	1.06 (0.43–2.66)			
	-	42	1	0.88		
Malignant disease	+	25	1.87 (0.73–4.51)			
	-	66	1	0.18		
SGA	A	53	1.33 (0.54–3.42)			
	B-C	38	1	0.53		
Sarcopenia	+	69	6.11 (1.25–111.0)			
	-	22	1	0.02		
Sarcopenic obesity	+	31	3.52 (1.41–9.48)		2.72 (1.06–7.76)	
	-	60	1	<0.01	1	0.03

ASA-PS, American Society of Anesthesiologists physical status; CI, confidence interval; DCF, docetaxel, 5-fluorouracil and cisplatin; DM, diabetes mellitus; FP, 5-fluorouracil and cisplatin; HR, hazard ratio; NAC, neoadjuvant chemotherapy; SGA, subjective global assessment.

**Table 4:** Characteristics of nutrition-related factors in elderly patients with or without sarcopenic obesity at baseline.

	Sarcopenic Obesity	Non-Sarcopenic Obesity	
Variables	(*n* = 31)	(*n* = 60)	*p*
Age (years)	73.6 ± 3.1	74.3 ± 2.9	0.36
Sex (male/female)	27/4	46/14	0.28
ASA-PS (1/2)	7/24	14/46	1.00
Body mass index (kg/m^2^)	22.2 ± 2.3	20.4 ± 2.8	<0.01
Body mass index >25 kg/m^2^	3 (9.7)	3 (5.0)	0.40
Visceral fat mass (cm²)	134.7 ± 36.8	75.0 ± 53.3	<0.01
Albumin (g/dL)	4.10 ± 0.36	4.05 ± 0.44	0.21
CCr	74.4 ± 12.5	74.1 ± 16.4	0.92
ChE	307 ± 70	281 ± 54	0.06
T-chol	188.8 ± 40.1	189.9 ± 28.5	0.88
Hb	13.8 ± 1.1	13.3 ± 1.3	0.06
SGA (A/B or C)	26/5	27/33	<0.01
Body weight loss rate (%)	1.7 ± 3.2	4.3 ± 6.2	0.03
%VC	98.6 ± 16.2	101.6 ± 14.6	0.38
%FEV1.0	77.0 ± 6.9	76.8 ± 8.3	0.90
Diabetes mellitus, *n* (%)	7 (22.6)	13 (21.7)	1.00
Cardiovascular disorder, *n* (%)	17 (54.8)	32 (53.3)	1.00
Cerebrovascular disorder, *n* (%)	1 (3.2)	1 (1.7)	1.00
Malignant disease, *n* (%)	9 (29.0)	16 (26.7)	0.81
Chronic disease, *n* (%)	3 (9.7)	7 (11.6)	1.00

Data are expressed as the number of cases or mean number ± standard deviation. ASA-PS, American Society of Anesthesiologists physical status; %VC, percentage vital capacity; %FEV1.0, percentage forced expiratory volume in 1 s; CCr, creatinine clearance; ChE, cholinesterase; T-chol, total cholesterol; Hb, hemoglobin; SGA, subjective global assessment.

**Table 5:** Treatment-related factors in elderly patients with and without sarcopenic obesity.

	Sarcopenic Obesity	Non-Sarcopenic Obesity	
Variables	(*n* = 31)	(*n* = 60)	*p*
Adverse events (≥ grade 3), *n* (%)			
Hematological			
Neutropenia	11 (43.2)	29 (58.3)	0.49
Non-hematological adverse events			
Diarrhea	0 (0)	3 (3.8)	0.54
Malaise	2 (6.8)	6 (6.8)	1.00
Anorexia	2 (6.8)	6 (6.8)	1.00
Incomplete case	2(6.5)	4(6.7)	1.00
Operation time (min)	522.7 ± 92.6	484.0 ± 96.2	0.06
Hospital stay (days)	33.4 ± 20.9	26.8 ± 15.8	0.09
Surgery related complications (≥grade 3), *n* (%)	13 (41.9)	16 (26.7)	0.15
Anastomotic	3 (9.7)	1 (1.7)	0.11
Pneumonia	5 (16.1)	2 (3.3)	0.04
Recurrent nerve paralysis	1 (3.2)	1 (1.7)	1.00
SSI	3 (9.7)	7 (11.7)	1.00
Chylothorax	1 (3.2)	1 (1.7)	1.00
Arrhythmia	0 (0)	0 (0)	1.00
Anastomosis stenosis	8 (25.8)	7 (11.7)	0.13

Data are expressed as the number of cases or mean number ± standard deviation. SSI, surgical site infection.
